# Resin-based dental pulp capping restoration enclosing silica and portlandite nanoparticles from natural resources

**DOI:** 10.1038/s41598-024-66728-0

**Published:** 2024-07-17

**Authors:** Mai M. Elbatanony, Engie M. Safwat, Sammar El-Sherif, Mohammad L. Hassan

**Affiliations:** 1 Restorative and Dental Materials Department, Oral and Dental Research Institute, National Research Centre, Giza, Egypt; 2 Cellulose and Paper Department, Centre of Excellence for Advanced Sciences, National Research Centre, Giza, Egypt

**Keywords:** Remineralizing pulp capping, Rice husk, Silica, Carbonated lime, Portlandite, Sugar beet, Dental biomaterials, Biomineralization

## Abstract

Natural-based materials represent green choices for biomedical applications. In this study, resin pulp capping restoration enclosing strengthening silica and bioactive portlandite nanofillers were prepared from industrial wastes. Silica nanoparticles were isolated from rice husk by heat treatment, followed by dissolution/precipitation treatment. Portlandite nanoparticles were prepared by calcination of carbonated lime waste followed by ultrasonic treatment. Both were characterized using x-ray diffraction, energy dispersive x-ray, and transmission electron microscopy. For preparing pulp capping restoration, silica (after silanization) and/or portlandite nanoparticles were mixed with 40/60 weight ratio of bisphenol A-glycidyl methacrylate and triethylene glycol dimethacrylate. Groups A, B, and C enclosing 50 wt.% silica, 25 wt.% silica + 25 wt.% portlandite, and 50 wt.% portlandite, respectively, were prepared. All groups underwent microhardness, compressive strength, calcium release, pH, and apatite forming ability inspection in comparison to mineral trioxide aggregate (MTA) positive control. In comparison to MTA, all experimental groups showed significantly higher compressive strength, group B showed comparable microhardness, and group C showed significantly higher calcium release. Groups B and C showed prominent hydroxyapatite formation. Thus, the preparation of economic, silica-fortified, bioactive pulp capping material from under-utilized agricultural residues (rice husk) and zero-value industrial waste (carbonated lime from sugar industry) could be achieved.

## Introduction

The need to preserve the vitality of dental pulp in traumatized or carious teeth introduced the concept of designing vital pulp therapy. In this context, a ‘direct pulp capping’ procedure is performed, where the exposed vital pulp is cured using a special restorative material that should help to heal while maintaining the protection and vitality of the dental pulp^1^.

Calcium hydroxide has been widely used in direct pulp capping treatment processes^2^. It is the most appropriate material for this purpose due to its superior ability to induce dentin bridge formation^3^. However, direct pulp capping using calcium hydroxide might negatively affect pulp tissue vitality because of its strong alkalinity which might result in a wide necrotic layer formation on top of the pulp tissue^4^. In addition, calcium hydroxide lacks adhesion to tooth structure, has inadequate mechanical strength, and is soluble in an acidic environment^5^. The dissolution of calcium hydroxide can cause microleakage within 1–2 years of its application, with subsequent necrosis and infection of approximately 50% of the treated pulps^2^.

Mineral trioxide aggregate (MTA) had surpassed calcium hydroxide as direct pulp restoration, owing to its excellent clinical results^1,6^. MTA is a calcium silicate-based cement that is recommended for pulp capping therapy, root end, and root canal perforation restoration^7,8^. It has excellent biocompatibility, antibacterial properties, and bone induction capability, thanks to its calcium ions releasing property^9^. MTA can chemically bond to hard tooth structure forming an intimate hermetic seal^10^. However, MTA suffers from various drawbacks such as difficulty in handling, high cost, high solubility, and prolonged setting time^11^.

An ideal direct pulp capping material should be simple to handle clinically and can promote dentin bridge formation while adhering tightly to dentin to prevent microleakage with subsequent bacterial invasion^12,13^.

With the recent global dental esthetic revolution, dental resin composite restoration has been widely utilized as a direct esthetic restorative material in the oral cavity, owing to its natural esthetic color, high strength, strong adhesion to the tooth structure and short controllable setting time^14^.

When direct pulp capping treatment is performed using resinous restoration, pulp preservation is likely achieved. This concept was supported by the results of many studies^14,15^ that showed good pulp healing in resin-capped teeth. Other studies added that even though there was no adverse effect when resinous restoration was applied directly on the pulp, the dentin bridge formation took significantly longer time than that formed after calcium hydroxide application, this delay may expose the pulp to bacterial invasion through the resin dentin interface^16,17^.

Current studies on dental resin-based composites have been focusing on their filler content, type, and size. Silica has long been known as a common filler type in resin composite restorations, as it enhances their mechanical strength properties and lowers their water absorption^18,19^.

Silica is a naturally occurring mineral, it is the major constituent of sand, besides, it is naturally present in many agricultural residues such as rice husk and straw; it has a wide range of industrial and technological applications as a reinforcing agent and as a filler. Rice husk is considered a low-cost, sustainable renewable source of silica; it is composed of a mixture of cellulose, lignin, silica, alkalis, and trace elements. It has an ash content of 10–20%, of which 87–97% is silica. Currently, the demand to extract silica from rice husk through efficient and sustainable methods is increasing^20^.

The use of silica nanoparticles from rice husk to improve the mechanical properties of dental materials started to drive scientists’ attention, due to its well-proven reinforcing characteristics^21^, they even used it as a method of increasing the hydroxyapatite formation in dentin and as an antimicrobial agent against Streptococcus mutans. Surprisingly, it proved efficient as a bioactive and antimicrobial agent^22^.

A systematic review discussing dental composite resins derived from rice husk biowaste concluded that rice husk dental composite could be a promising alternative to conventional dental composites, thanks to its low cost, eco-friendliness, and acceptable clinical performances^23^.

Portlandite, on the other hand, is a hydrated calcium oxide that can be considered as dentin-promoting agent due to its calcium-releasing ability^24^. It is also highly recommended due to its low cost and biocompatibility. During sugar extraction from sugar beet, calcium hydroxide is used to raise the pH of the juice and to react with the impurities and the coloring materials produced during sugar refining. Upon addition of carbon dioxide to the aforementioned mixture, insoluble calcium carbonate precipitates enclosing the impurities and the coloring materials, which can be removed by filtration^25^^.^ The carbonated sludge, ‘also known as carbonated mud’, produced from sugar industrial sludge, is estimated to be over thirty million tons, worldwide, per year^26^. This waste has been utilized in different applications and products such as for clarification of raw sugar^27^, in cement products^28^, in bio-fertilizers and bio compost^26,29^, limestone-gypsum desulfurization^30^ animal feed mixtures, and as a filler in paper^31^, plastic^32^, and adhesives^33^.

In the current study, a resinous dental pulp capping material was prepared by incorporating environment-friendly silica nanoparticles extracted from rice husk and portlandite nanoparticles obtained from carbonated mud waste into a light-curable resinous matrix. Our aim was to achieve a new low-cost bioactive pulp capping material equivalent in bioactivity to MTA but with better mechanical and handling properties owing to its resinous content, without compromising its bioactive properties.

## Materials and methods

Rice husk was obtained from local farms in Qalubiayah governorate, Egypt. It was washed with water and left to air-dry. The chemical composition of rice husk was: 18.6% ash content, 47.6% α-cellulose, 12.5% pentosans, 22.9% klason lignin, and 3.7% ethanol-toluene extractives.

Carbonated mud was kindly supplied by ‘Alnubariah Company for Sugar’, in Alnubariah, Egypt. The clay was washed with distilled water until the effluent water had a neutral pH, and then it was dried in an oven at 105 °C for 4 h before use.

### Preparation and characterization of silica nanoparticles from rice husk

Silica was prepared by heat treatment of rice husk at 800 °C for 1 h in a muffle furnace. After the sample was cooled to room temperature, the isolated silica was washed using distilled water till the neutral pH of water filtrate and dried in a vacuum oven at 105 °C for 2 h. The isolated silica was then dissolved in 0.1 N NaOH followed by precipitation by drop-wise addition of dissolved silica in 0.1 N HCl under vigorous stirring at room temperature. The prepared sample was collected by centrifugation at 10,000 rpm, purified by repeated washing using distilled water, and centrifuged. Finally, the suspended silica nanoparticles were dialyzed against distilled water until neutral pH.

The isolated silica nanoparticles were characterized using a transmission electron microscope (TEM) using a JEOL TEM (JEM-2100, JEOL, Tokyo, Japan) with an acceleration voltage of 100 kV. A drop of the suspension was used on a copper grid bearing a carbon film. X-ray diffraction pattern (XRD) was determined using a Bruker diffractometer (Bruker D 8 advance target). Cu Kα radiation source with a second monochromator (λ = 1.5405Ǻ) at 40 kV and 40 mA was used and the scanning rate was 0.2 min^−1^.

The atomic percentages of the prepared silica nanoparticles were obtained using Energy dispersive X-ray spectroscopy (EDX), hyphenated with Quanta FEG 250 scanning electron microscopy. The spectra were displayed on TEAM ® software at the acceleration voltage 20 kV.

### Preparation and characterization of portlandite nanoparticles from carbonated mud waste

Nano-hydrated calcium oxide (portlandite) was prepared by heat treatment of the carbonated dry mud waste at 800 °C for 1 h in a muffle furnace. After the sample was cooled to room temperature, it was washed with distilled water until neutral pH of water filtrate then dried in a vacuum oven at 105 °C for 2 h. To obtain the portlandite nanoparticles, the dried sample was suspended in distilled water (1 g in 50 ml of water) and ultrasonicated using Hielscher ultrasonic processor (Hielscher Ultrasonics GmbH, Germany) for 5 min; a 1-cm diameter probe was used and amplitude was set at 75% of the maximum. During the ultrasonic treatment, the temperature of the suspension was cooled down using ice, to avoid water evaporation. The prepared portlandite nanoparticles were characterized using TEM, EDX, and XRD as mentioned above for silica nanoparticles.

### Preparation of the resin matrix

The resin matrix was prepared by mixing 40 wt.% bisphenol A-glycidyl methacrylate (Bis-GMA, Sigma Aldrich, USA), and 60 wt.% triethylene glycol dimethacrylate monomers (TEGDMA, Sigma Aldrich, USA). For preparing the photo-initiator system, 0.5 wt.% camphorquinone (Sigma Aldrich, USA) and 0.5 wt.% ethyl 4-dimethyl-amino benzoate (EDAB, Sigma Aldrich, USA) were weighed, then added gradually to the prepared matrix and stirred for 1 h^34^.

### Silanization of the silica nanoparticles

Silane coupling agent was prepared by proportioning 70 wt.% ethanol in a glass beaker, a few drops of acetic acid were then added gradually into the solution to decrease the pH to 3–4. Finally 6 wt.% trimethoxysilane was added^11,35^. Stirring was done using a magnetic stirrer for 1 h. The silica nanoparticles synthesized from rice husk were incubated in the silane coupling agent for 2 h and then centrifuged (Megafuge 8R, Thermo Fisher, Germany) for 30 min. Finally, the precipitate was dried for 24 h in a hot oven at 80 °C^34^.

### Incorporation of the silanized silica and portlandite nanoparticles into the prepared resin matrix

The silanized silica and portlandite nanoparticles were incrementally added to the experimentally prepared resin matrix then they were hand mixed using a plastic spatula to form a homogenous resin mix in three different groups with a total 50% weight percentage loading. The prepared groups were designed as follows: Group A: 50 wt.% silanized silica nanofillers, group B: 25 wt.% silanized silica nanofillers and 25 wt.% portlandite nanofillers and group C: 50 wt.% portlandite nanofillers.

The three experimental groups A, B, and C were compared to a commercially available MTA product (PPH CERKAMED, Poland, batch number; 205211) supplied in the form of powder that consists of calcium oxide with oxides of silicon, iron, aluminum, sodium, potassium, bismuth, magnesium, zirconium and calcium phosphate. The mixing liquid was distilled water.

### Testing the mechanical properties of the prepared pulp capping materials

#### Compressive strength measurement

Five specimens for each group were prepared for compressive strength testing using cylindrical Teflon molds 4 mm in diameter and 6 mm in height according to ADA specification no. 27^36,37^. Each specimen was incrementally packed in a mold placed over a glass slab and a celluloid strip. Another celluloid paper was pressed at the mold’s top against another glass slab to extrude excess material. Curing was done using a light-emitting diode (LED) curing unit (RTA, MINIS, Guilin Woodpecker Medical Instrument Co. Ltd., China): 1000–1200 mW/cm^2^ for 40 s. Excess material was removed using Soflex discs. Compressive strength testing was done after immersion of the specimens in distilled water for 24 h using a universal testing machine (AGX- PLUS, SHIMADZU, 5KN) with 50 N load cell and crosshead speed 0.5 cm/min^38^.

#### Vickers microhardness measurement

Five specimens were prepared for each group using split Teflon molds of 5 mm in diameter and 2 mm in thickness^39^. Specimens were prepared as those prepared for compressive strength testing. Excess material was removed using Soflex discs. Hardness testing was then performed after immersion of the specimens in distilled water for 24 h using a Vickers hardness tester (NEXUS 4000 ™, INNOVATEST, model no.4503, Netherlands). Specimens were subjected to 100 g force for 15 s dwell time for each indentation^40^.

### Testing the bioactivity of the prepared pulp capping materials

#### Calcium ions-releasing ability and pH measurement

Five cured cylindrical specimens with dimensions of 4 mm in diameter and 6 mm in height were prepared as previously mentioned. The specimens were then immersed in a freshly prepared artificial saliva (2.38 g Na_2_HPO_4_, 0.19 g KH_2_PO_4,_ and 8.00 g NaCl per liter of distilled water adjusted with phosphoric acid to pH 6.75)^35^ and stored at 37 °C for 1, 7 and 14 days. The volume of the artificial saliva was adjusted to be 10 mm^3^ according to the equation Vs = Sa/10, where Vs is the volume of the artificial saliva, and Sa is the apparent surface area of each specimen^41^. The calcium ions concentration was measured by an inductively optical emission spectrometer (Ultima Exports, HORIBA, France). pH was measured after each incubation period using a pH meter (Jenway 3505, Bibby Scientific Limited, UK).

#### Evaluating the apatite-forming ability of the prepared groups

Five cured cylindrical specimens with dimensions of 4 mm in diameter and 6 mm in height were prepared and stored in 10 mm^3^ of artificial saliva for 14 days^41^. The specimen surfaces were characterized using scanning electron microscopy and energy dispersive X-ray spectrometry (SEM–EDX; JCM-6000Plus NeoScopTM, JEOL Ltd., Tokyo, Japan). The EDX analysis was carried out on the surface of the crystals present in the SEM image of each group. Two locations were selected and the results of the EDX analysis were averaged.

### Statistical analysis

Numerical data were represented as mean and standard deviation (SD). Shapiro–Wilk's test was used to test for normality. Homogeneity of variances was tested using Levene's test. Data showed parametric distribution and variance homogeneity. Mechanical properties data were analyzed using one-way ANOVA followed by Tukey’s post hoc test. Calcium ions release and pH data were analyzed using two-way mixed model ANOVA. Comparisons of simple effects were done utilizing one-way ANOVA followed by Tukey’s post hoc test for independent variables and repeated measures ANOVA followed by Bonferroni post hoc test for repeated measurements. P-values were adjusted for multiple comparisons using Bonferroni correction. The significance level was set at p < 0.05 within all tests. Statistical analysis was performed with R statistical analysis software version 4.2.3 for Windows. (R Core Team (2023) (R Foundation for Statistical Computing, Vienna, Austria. URL https://www.R-project.org/.)

## Results

### Characterization of silica nanoparticles prepared from rice husk

**Figure 1:**
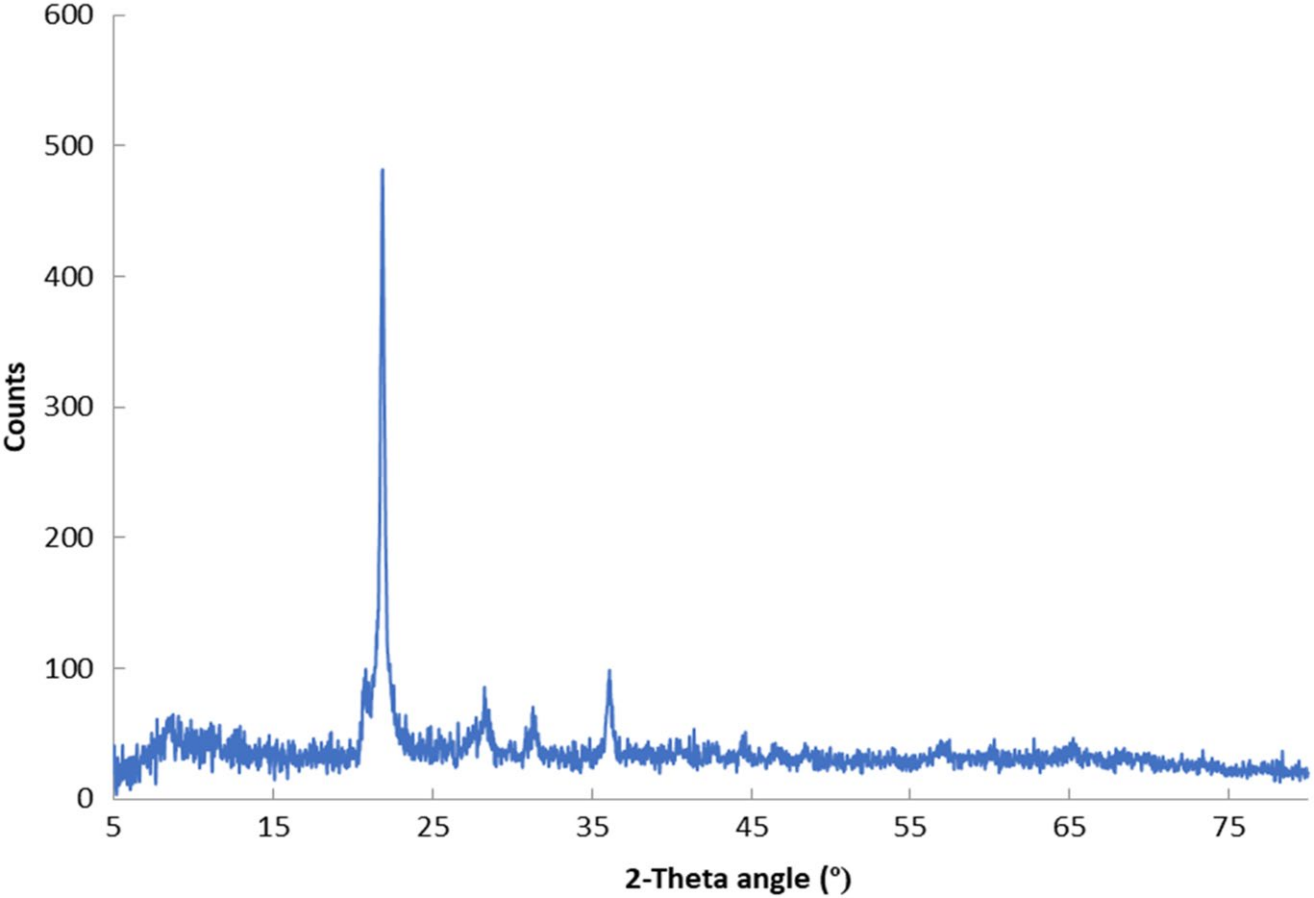
The XRD pattern of the prepared rice husk silica nanoparticles.

The prepared silica nanoparticles were characterized using XRD, TEM, and EDX. The XRD pattern of the prepared silica nanoparticles presented in Fig. 1, showed that they had cristobalite structure with reflection peaks at 2θ values of 21.7°, 28.4°, 31.3° and 36.1°, which correspond to 101, 111, 102, and 200 crystal planes^42^.

**Figure 2:**
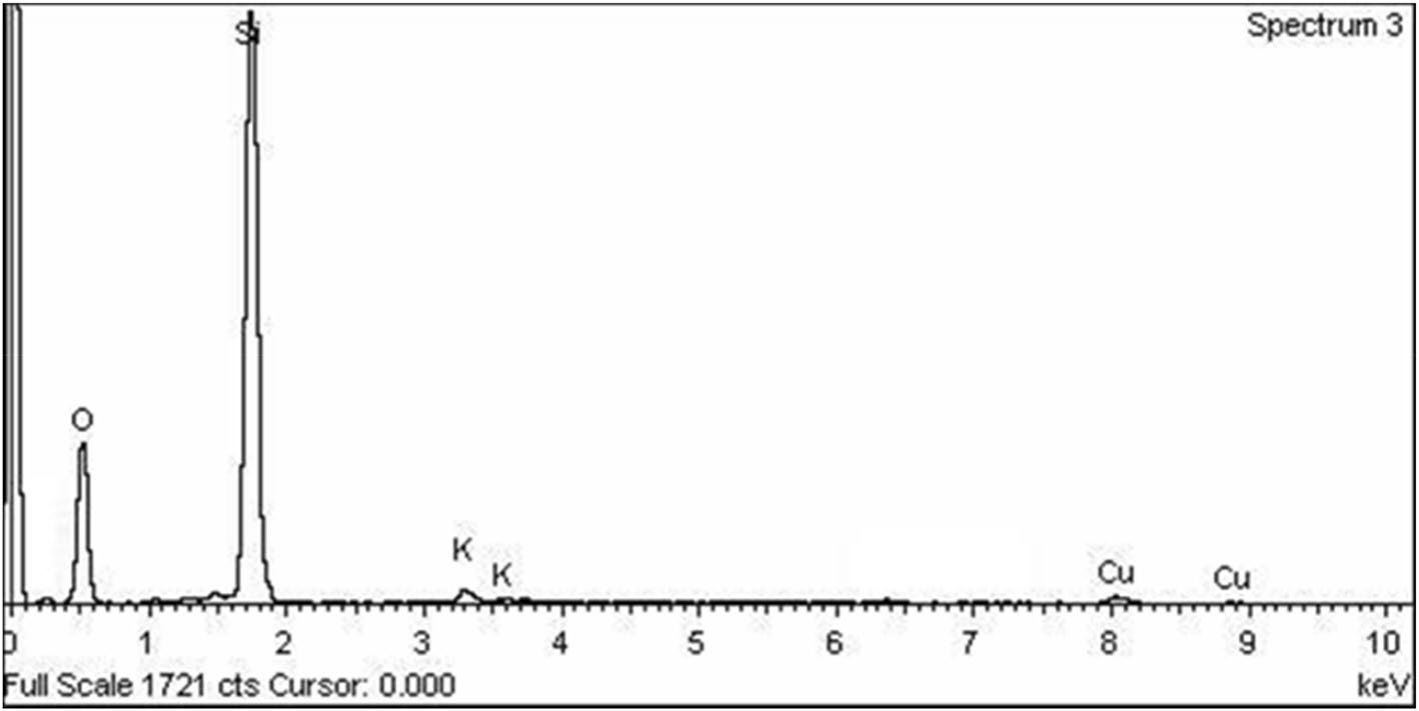
The EDX analysis of the prepared silica nanoparticles.

The EDX analysis, shown in Fig. 2, revealed the purity of the prepared silica nanoparticles since Si and O atoms had peaks with strong intensity with traces of Cu and K. The sum of the atomic percent of silicon and oxygen atoms was 99.46, while that of Cu and K together was 0.53%.

**Figure 3:**
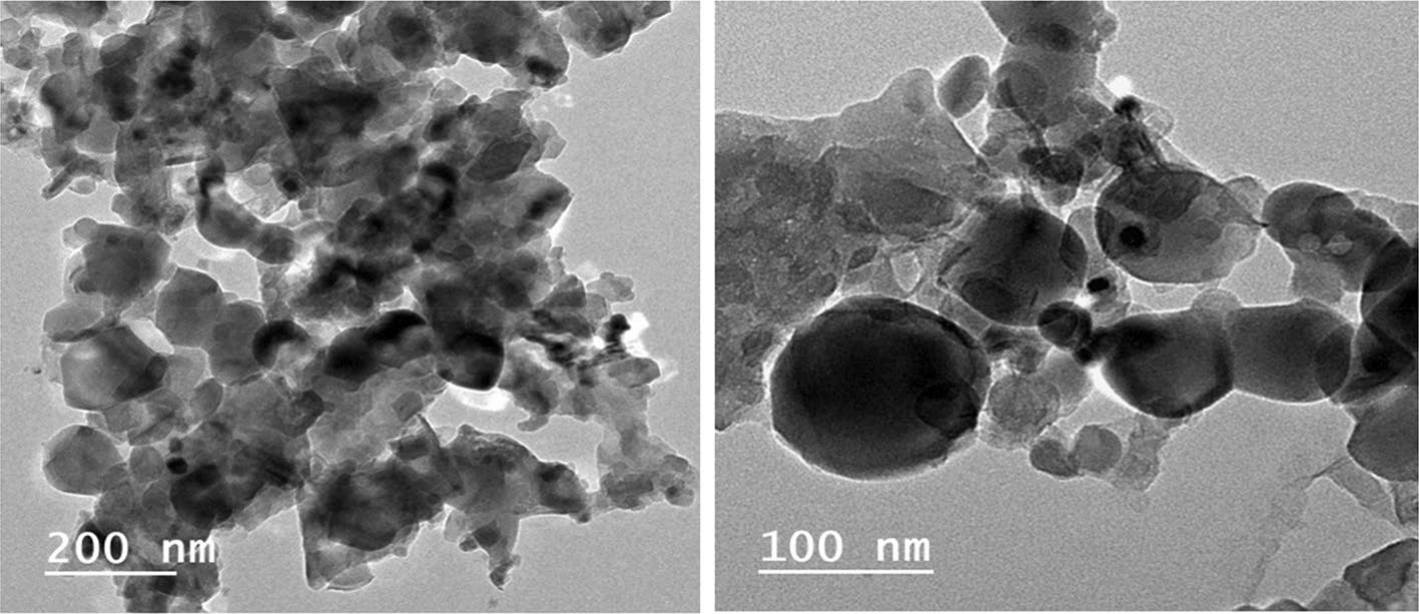
The TEM images of the prepared silica nanoparticles at different magnifications.

Regarding the size of the prepared silica nanoparticles, TEM images showed particle size with a diameter range of 23–116 nm (Fig. 3).

### Characterization of portlandite nanoparticles prepared from carbonate clay waste

**Figure 4:**
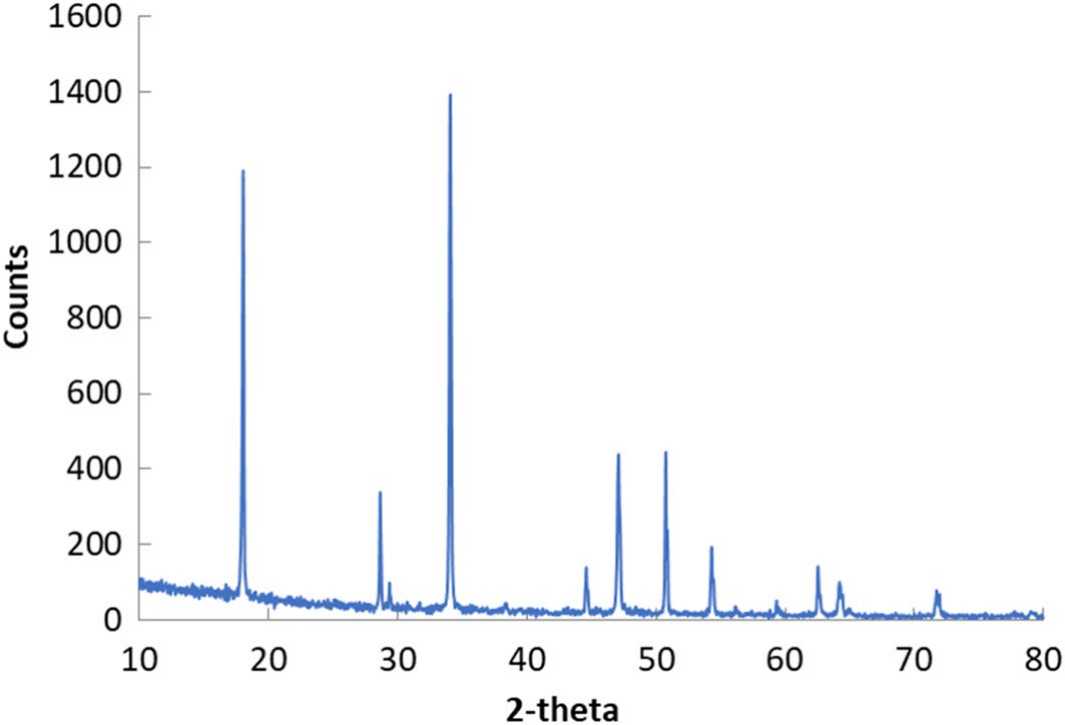
The XRD pattern of the prepared portlandite nanoparticles.

Figure 4 presented the XRD pattern of the prepared hydrated calcium oxide. The pattern showed that the prepared particles had portlandite crystal structure ((Ca(OH)_2_; ICDD card No. 00-044-1481) with peaks at 2θ values of 18.0, 28.6, 34.0, 44.6, 47.1, 50.7, 54.3, 62.5, 64.2, and 71.6 which correspond to reflection from crystal planes of 111, 110, 112, 101, and 102^43,44^.

**Figure 5:**
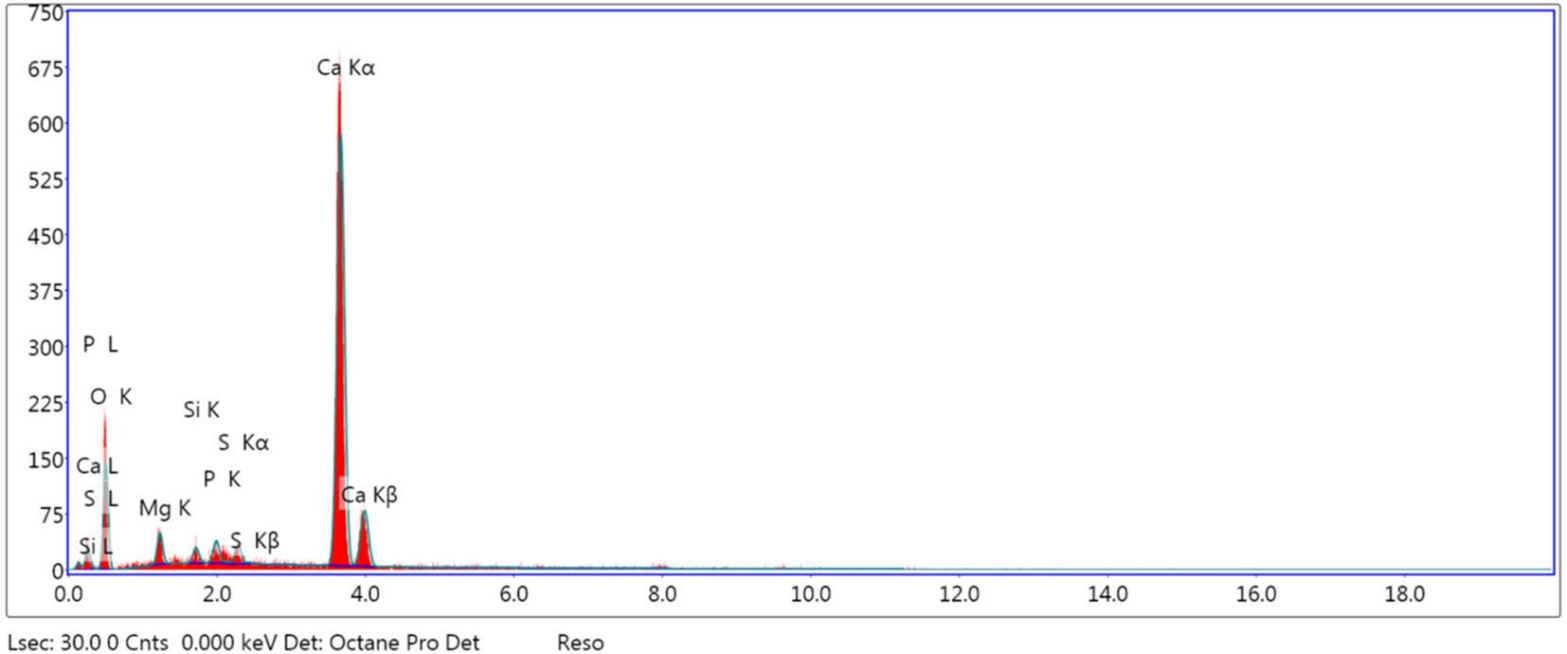
The EDX analysis of the prepared portlandite nanoparticles.

The EDX analysis shown in Fig. 5 displayed the good purity of the prepared portlandite nanoparticles, since the peaks for Ca and O atoms predominated in the spectrum. Traces of magnesium, sulfur, phosphorus, and silicon, which could have originated from the carbonated mud^27,28^, were also found.

**Figure 6:**
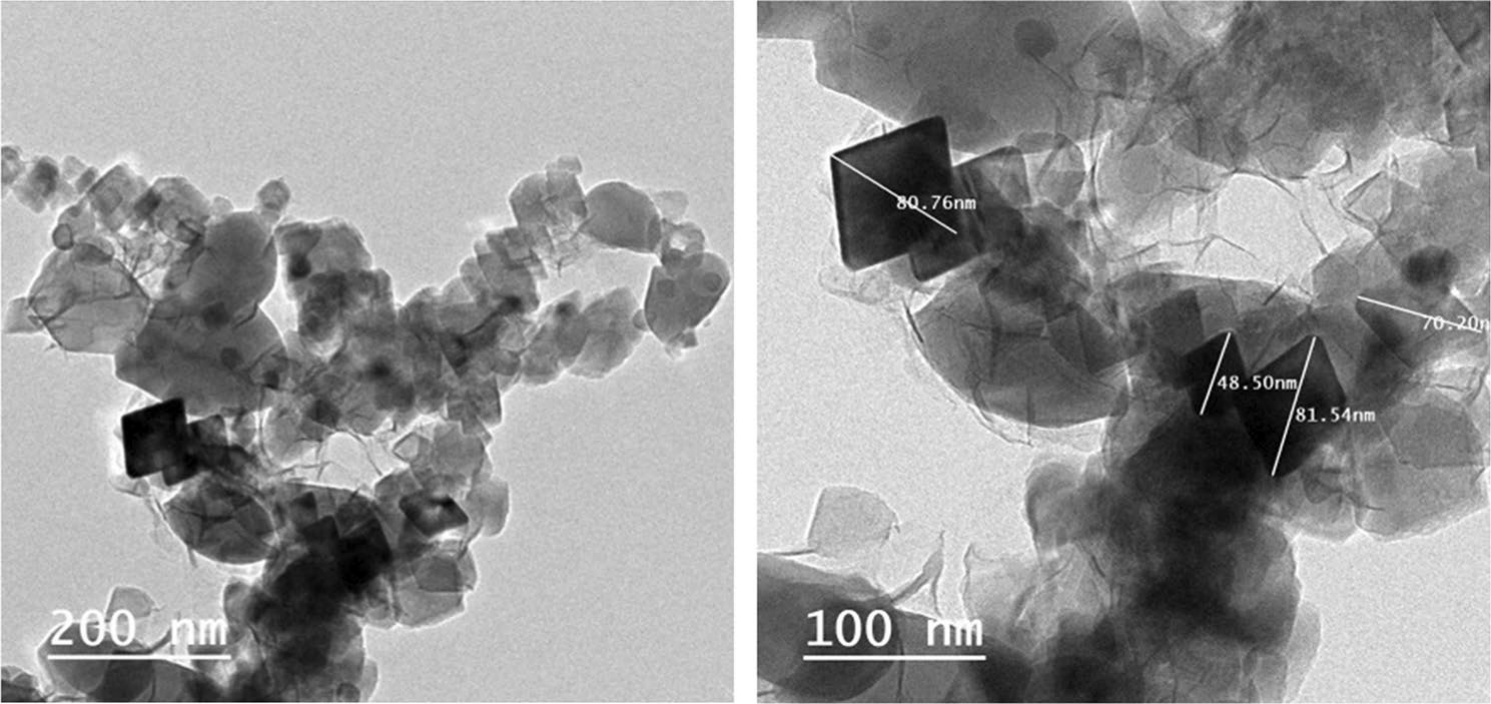
The TEM images of portlandite nanoparticles at different magnifications.

The TEM images presented in Fig. 6 on the other hand, revealed the tetragonal and hexagonal plate-like shape of the prepared portlandite nanoparticles with dimensions ≤ 100 nm.

### Mechanical properties evaluation of the prepared pulp capping materials

Intergroup comparisons of the mechanical properties of the experimentally prepared pulp capping material are presented in Table 1. The proportion of the components in each group was determined according to preliminary experiments to obtain samples with reasonable strength and handling properties.

**Table 1:** The intergroup comparison of the tested mechanical properties.

Measurement	(Mean ± SD)				f-value	p-value
	Positive control	Group A	Group B	Group C		
Max. compressive stress (MPa)	2.37 ± 0.14^B^	87.03 ± 17.57^A^	95.79 ± 24.96^A^	100.88 ± 42.50^A^	13.05	< 0.001*
Max strain (%)	11.27 ± 3.93^A^	15.64 ± 6.76^A^	13.30 ± 4.75^A^	11.90 ± 4.75^A^	0.86	0.476
Modulus of elasticity (MPa)	59.78 ± 29.49^B^	1347.77 ± 250.47^A^	1392.42 ± 11.35^A^	1630.75 ± 380.44^A^	40.46	< 0.001*
Hardness (VHN)	55.24 ± 4.90^A^	35.20 ± 12.47^B^	44.28 ± 10.22^AB^	35.46 ± 2.14^B^	5.69	0.006*

Different superscript letters indicate a statistically significant difference within the same horizontal row; *significant (p < 0.05). Group A: 50 wt.% silanized silica nanofiller, group B: 25 wt.% silanized silica and 25 wt.% portlandite nanofillers, and group C: 50 wt.% portlandite nanofiller.

The results of the mechanical properties evaluation showed that; for maximum compressive strength and modulus of elasticity, there was a significant difference between the tested groups, with the positive control (MTA), where MTA had a significantly lower value than all the experimental groups (p < 0.001). However, the maximum strain results showed no statistically significant difference among all groups at p = 0.476.

As for the hardness results, the positive control group revealed significantly higher values than those of groups A and C, though there was no significant difference in the hardness values of group B.

### Calcium ions release

Results of the mixed model analysis of the calcium ions release data revealed a significant interaction between the groups and the time of measurement (p < 0.001). Comparison of simple effects showed that; for different time intervals, all tested groups revealed significant differences in-between (p < 0.001). On day 1, post hoc pairwise comparisons displayed significantly higher calcium ions release for the positive control group than for the other groups (p < 0.001). At day 7, group C had significantly higher calcium ions release than the other groups (p < 0.001). In addition, the positive control and group B had significantly higher values than group A (p < 0.001). At day 14, all pairwise comparisons were statistically significant (p < 0.001), with group C having the highest calcium ions release, followed by the positive control, then group B and group A having the lowest mean value.

**Figure 7:**
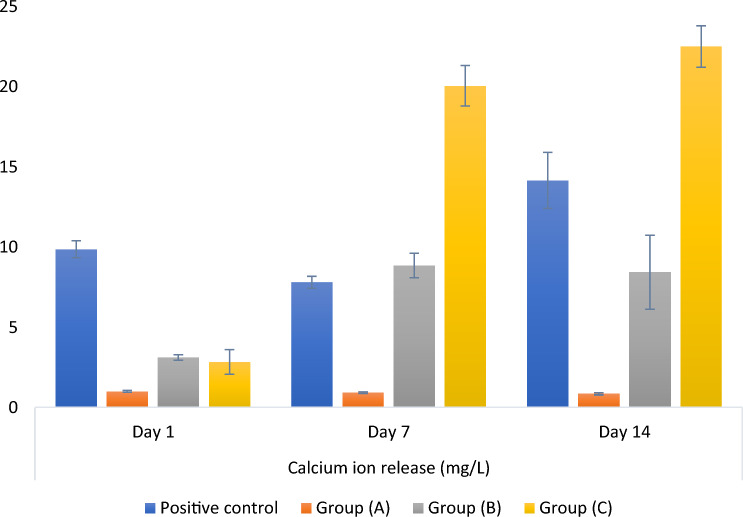
Bar chart representing mean and standard deviation values for calcium ions release (mg/L). (Group A: 50 wt.% silanized silica nanofiller, group B: 25 wt.% silanized silica and 25 wt.% portlandite nanofillers, and group C: 50 wt.% portlandite nanofiller).

For the positive control group, calcium ions release after 7 days was significantly lower than that after 1 and 14 days (p < 0.001), while for group A, the difference was not statistically significant (p = 0.105). For groups B and C, the difference was statistically significant, with calcium ions release values measured after 7 and 14 days being significantly higher than that at day 1 (p < 0.001). Mean and standard deviation values for calcium ions release are presented in Fig. 7.

### pH analysis

The results of the mixed model analysis (p = 0.006) of the pH data revealed that there was a significant interaction between the groups and the time of measurement. Comparison of the simple effects showed that; for days 1 and 7, there was a significant difference between the tested groups (p < 0.05), while for day 14, the difference was not statistically significant (p = 0.333). On day 1, post hoc pairwise comparisons showed that the mean pH values of group C were significantly lower than those of the other groups (p < 0.001). On day 7, the positive control group exhibited a significantly higher mean pH value than the other groups (p < 0.001).

**Figure 8:**
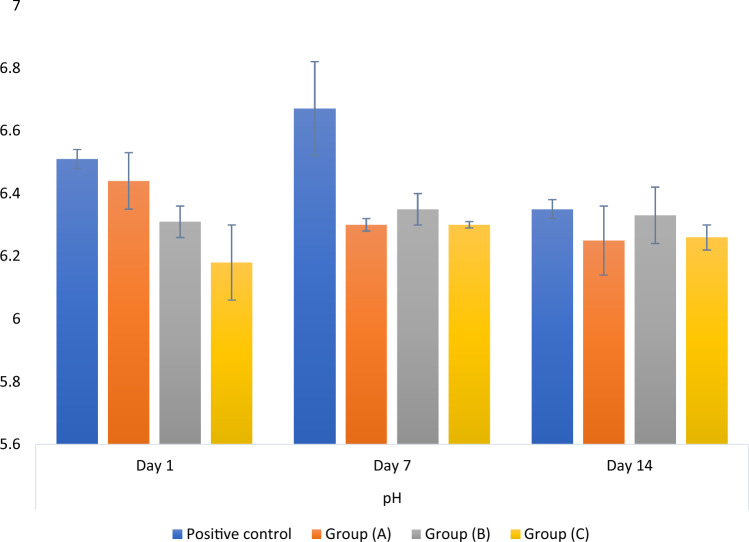
Bar chart showing mean and standard deviation values for pH. (Group A: 50 wt.% silanized silica nanofiller, group B: 25 wt.% silanized silica and 25 wt.% portlandite nanofillers, and group C: 50 wt.% portlandite nanofiller).

For the positive control group, pH values measured after 7 days were significantly higher than those measured after 14 days (p = 0.030). While for the other groups, there was no statistically significant difference in their pH values among different time intervals (p > 0.05). Figure 8 represents the mean and standard deviation of the recorded pH values.

### The apatite forming ability

**Figure 9:**
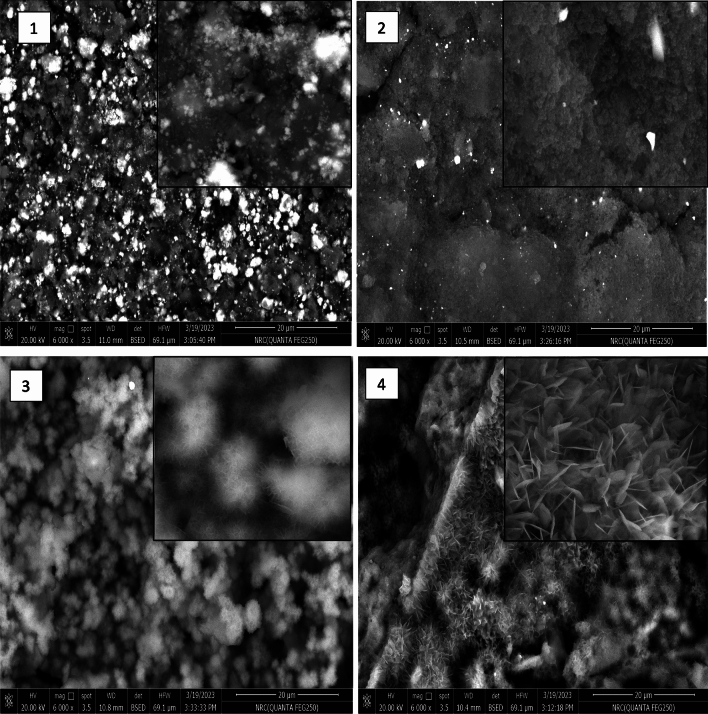
The SEM images of 1: MTA positive control, 2: group A (50 wt.% silanized silica nanofiller), 3: group B (25 wt.% silanized silica + 25 wt.% portlandite nanofillers), and 4: group C (50 wt.% portlandite nanofiller), at magnification 6000 × and 24,000 × .

**Figure 10:**
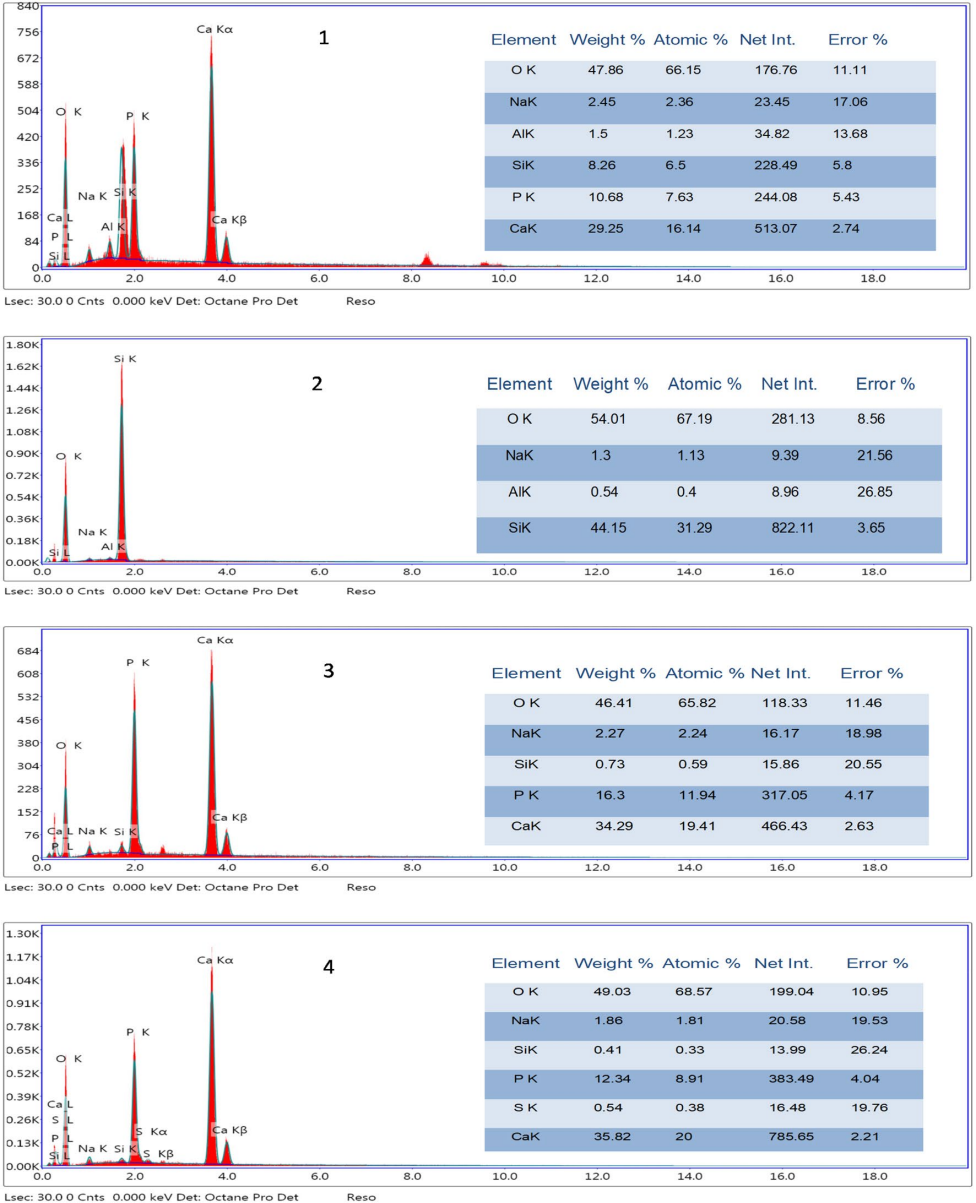
The EDX analysis of 1: MTA positive control, 2: group A (50 wt.% silanized silica nanofiller), 3: group B (25 wt.% silanized silica + 25 wt.% portlandite nanofillers) and 4: group C (50 wt.% portlandite nanofiller).

The SEM images as well as the results of the EDX analysis are presented in Fig. 9 and Fig. 10 respectively, for the positive control and the experimental groups, after soaking in artificial saliva for 14 days.

As revealed in the SEM images, there was evidence of the formation of calcium phosphate deposits in the positive control group, group B, and group C, these deposits had irregular morphology in the positive control group, shuttle/flower-like shape in group B, and nano-spherulites packed as clusters of spheroidal masses having needle-like projections in group C.

On the other hand, group A showed the absence of formation of any calcium phosphate deposits.

These results were confirmed by the data obtained from the EDX analysis, where, the Ca/P atomic ratio of the presented calcium and phosphorous percentages were 2.11, 0, 1.63, and 2.25 for the positive control, group A, B, and C respectively.

## Discussion

Pulp capping therapy is a well-known restorative procedure aimed to preserve the vitality of the restored teeth; however, it represents a big challenge to dental practitioners and consequently, manufacturers owing to its proximity to the sensitive pulp tissue.

In the current study, two nanoparticles have been prepared for use in pulp capping restorations. The preparation processes involved several steps. In the case of portlandite nanoparticle preparation, the process was as follows: (1) Heat treatment at 800 °C, which is known industrially as calcination and is used in several industries, such as preparation of high-quality fillers. Calcination is a closed process as all heat and gases evolved are utilized and carefully recycled^45^. In our study, calcination caused the evolution of carbon dioxide gas in a relatively pure form and thus could be easily collected from the system to be used as a gas or in other industrial operations. (2) Ultrasonic treatment of the produced portlandite to form nano-size particles. Ultrasonic technology is a green technology for the preparation of nanomaterials, as it does not involve chemical utilization and is not a high-energy consuming technology; moreover, ultrasonic technology has potential applications in our daily life activities like household washing^46^.

In the case of silica nanoparticle, the same heat treatment mentioned above was used, and then the dissolution using sodium hydroxide followed by the precipitation using hydrochloric acid was carried out. Heat treatment of rice husks generates energy that is usually used as a source of heating in several industries^47^. The alkali and acid used in the preparation process are commonly used in many industries such as the preparation of food-grade materials. For example, hydrochloric acid is used as an acidity regulator in the food industry (used under the symbol E507) while sodium hydroxide is used in curing olives and in removing the skins of fruits and vegetables to be canned^48^.

Regarding the raw materials of the existing commercially available pulp capping materials, the formulation may differ according to their type and manufacturer, some are green-based, as their main ingredient depends on refined Portland cement as ProRoot MTA (Dentsply tulsa, Johnson City, TN, USA) and TheraCal (Bisco Inc, Schaumburg, IL, USA). However, others are not green-based as their ingredient depends on synthetic chemical oxides as Ledermix MTA (Riemser, Riems, Germany), MTA Angelus (Angelus dental solutions, Londrina, PR, Brazil), BioAggregate (Innovative BioCeramix Inc, Vancouver, Canada)^49^, as well as, the current commercial product used as a positive control in this study (MTA, PPH CERKAMED, Poland).

The most used filler in dental composite resin restorations is silica. Silica particles of varying sizes and shapes can improve the physico-mechanical properties of dental composites. Most of the silica particles utilized in the resin composite synthesis are fabricated through the sol–gel process, using harmful and costly chemicals, like tetraethyl orthosilicate and sodium silicate. In the current study, silica was obtained from an inexpensive bio-based source; rice husk, and without the use of toxic chemicals. Bio-based materials are renewable, sustainable, and ecological^34,50^. Silica nanoparticles were prepared from rice husk in a cristobalite crystal structure, as the XRD pattern showed.

TEM image of the prepared silica confirmed their spherical shape and nano size (23–116 nm). The most preferred size and shape of the silica fillers for use in dental composite restoration fabrication are the nano, spherical fillers. These preferred characteristics allow increased filler loading thus enhancing composites’ fracture strength^51,52^. Moreover, small fillers with variable sizes allow denser packing, which raises the volume fraction of the fillers in the resinous composite restoration. All these features were fulfilled by the silica nanoparticles prepared in this study from rice husk; this could explain the good mechanical properties of the experimental resinous pulp capping materials^50^.

The incorporation of nanoparticles into the resin matrix improves its wear resistance, modulus of elasticity, flexural strength, tensile strength, and fracture toughness^19^; however, nanoparticles tend to agglomerate if their surface is not treated^51^. The agglomeration and lack of distribution in the resin matrix produce weak points as well as stress concentration areas, with the final failure of the restoration^19^. For this reason, the silica nanoparticles utilized in this study were silanized using 6 wt.% trimethoxysilane coupling agent. The silanization process of the silica nanoparticles hydrolyzes the (–OCH_3_) groups into silanol groups using water in the ethanol solvent. At that point, the silanol groups form a covalent bond with hydroxyl groups on the silica surface. The silanol groups on adjacent silanes condense together forming a polymer film on the silica surface. Furthermore, hydrogen bonds form between the hydroxyl groups on the surface of silica particles and the carbonyl groups of silane coupling agent via Vander Waals force^50,53^. Silanization of the silica nanoparticles provides a homogenous distribution of the nanofillers in the resinous restoration with an essential bond between both the matrix and the filler phases^50,51^. Due to the reasons mentioned above, adequate wetting of the prepared silica nanofillers was achieved in this study, with an overall improved performance of the material.

Previous studies mentioned that the formed dentin bridge after direct resinous pulp capping application was significantly slower than that obtained after direct calcium hydroxide application, thus microleakage may result, with subsequent failure^16,17^. Therefore, in this study, a new resinous system containing dentin-promoting agent (portlandite nanoparticles) was developed. This study matched the scope of other studies where Suzuki et al*.*^54^ studied the wound healing process of rats’ dental pulps that were directly pulp-capped via an experimental resinous restoration containing calcium phosphate as dentin dentin-promoting agent. The results of this study revealed that the incorporation of calcium phosphate into the experimental resinous restoration efficiently promoted dentin bridge formation and that the quantity of reparative dentin formed was directly related to the concentration of the added calcium phosphate. Another study prepared by Kato et al.^55^ reported that the experimental resinous pulp capping system containing dentin-promoting agents such as hydroxyapatite, brushite, whitlockite, and octacalcium phosphate, induced reparative dentin. Similarly, in the present study, nano hydrated calcium oxide (Ca(OH)_2_) (portlandite) was added as dentin-promoting filler to the experimental pulp capping restoration to generate a self-healing potential to the pulp and to reduce microleakage at the restoration-cavity interface. Pure portlandite with dimensions ≤ 100 nm was prepared from the carbonated clay waste of sugar beets that generated huge amounts of carbonation lime wastes during the industry process, holding calcium-rich portlandite nanoparticles.

The ultimate goal of pulp capping materials is reliable biological properties, thus calcium ions release, pH, and apatite forming ability inspection were performed to examine the new experimental pulp capping materials’ bioactivity.

However testing their mechanical and physical properties is no less important than testing biological properties, as any dental material intended to restore teeth should sustain the subjected masticatory and para-functional forces, accordingly inspecting the mechanical properties of a new dental restoration is an important factor in determining its clinical success^50^. Additionally, a serious drawback of most MTA restorations is their weak mechanical properties^9,11^, thus, in the current study both mechanical and biological properties were examined.

Regarding the mechanical properties evaluation, the effect of the filler type on the compressive strength of the experimental groups was evaluated using a universal testing machine according to ADA specification no. 27^37,38^. Results showed no statistically significant difference between the compressive strength results and the modulus of elasticity (stiffness) among the three experimental groups. As expected, the addition of silica increased the compressive strength values; however, good mechanical properties were achieved for all the inspected experimental groups.

Additionally, the compressive strength and the modulus of elasticity of the experimental groups were significantly higher than those of the positive control group (MTA). MTA is a calcium silicate-based material related to Portland cement with expected lower compressive strength when compared to a resinous pulp capping material enclosing rice husk silica and /or portlandite nanoparticles. The compressive strength of MTA is mainly affected by the size of the MTA powder, the mixing liquid, and the mixing techniques^56^. It was reported that MTA undergoes a hydration reaction with water resulting in calcium ions release with subsequent enhancement in its compressive strength. However, the depletion of the inorganic component from MTA may eventually decrease its compressive strength^2^.

Vickers hardness test is a reliable test to evaluate resinous composites. Generally, measuring the hardness of any dental restoration provides a good estimation of its wear resistance and stability against oral environmental changes^34,57^. Many factors can influence the hardness of the dental resinous composite restoration including, resin type, filler loading, and curing time^50,57^.

In the current study, the four tested groups exhibited reasonable hardness values, these values were comparable to a previous study^34^, in which, silica rice husk was incorporated in an experimental flowable resin composite, and their hardness results were between 29 and 31VHN.

Though groups A and C showed significantly lower hardness results than MTA (p < 0.001), yet, group B showed a comparable, non-significantly different result to MTA. Many studies^58,59^ suggested that Vickers hardness values could be increased by increasing the amount of special filler types such as zirconia, aluminum oxide, etc., these types of fillers were not added to the prepared experimental groups but are present in the MTA, where zirconia and aluminum are present in its composition as stated by the manufacturer.

Dental caries is a bothering dental disease that develops when the cariogenic bacteria metabolize carbohydrates, generating acids with a subsequent decrease in the pH value of saliva, resulting in teeth demineralization. A pH of 5.5–6 is known as the theoretically cariogenic pH^60^. The use of dental pulp capping materials that can release active remineralizing ingredients, such as calcium ions, is considered a smart strategy to stop dental caries.

In the current study, all recorded pH values at different immersion periods were above the critical cariogenic pH, this reveals a smart behavior of the investigated materials. This increase in the pH values occurs most likely because of the released cations originating from the tested materials.

The sustained release of calcium ions was evaluated by immersing the control and the experimental groups in artificial saliva for 1, 7, and 14 days. Some studies have inspected various immersion solutions, such as simulated body fluid, Hanks′ Balanced salt solution, Dulbecco's phosphate-buffered saline and distilled water; however, the current study utilized artificial saliva as an immersion medium to simulate the oral environmental conditions^61^.

The ability of MTA to exhibit high calcium ions release and alkalinize the surrounding fluids could be due to the surface hydration and dissolution of MTA calcium-silicate particles because of their high reactivity with water. This results in the formation of calcium hydroxide that dissolute into Ca and OH ions, which are released in the medium and elevate the pH^62–64^.

Though the experimental B & C groups have already calcium hydroxide (portlandite) in their filler structure that can readily release Ca and OH ions faster, yet, the pH of MTA was significantly higher than all the experimental groups on day 1 and 7. This could be explained by the fact that the experimental groups have resin in their composition, which may have regulated the Ca and OH ions release and consequently did not elevate the pH of the medium initially, however by the end of the immersion period (14 days) all groups showed comparable pH values.

Furthermore, the results of this study demonstrated that group B and group C pulp capping materials exhibited high levels of Ca ions release throughout the study. It is well known that amorphous calcium phosphate is initially formed in vivo then it transforms into a crystalline apatite-like phase by taking up OH^-^ ions from the solution^65–67^.

Calcium silicate-based restorations could have a sealing effect, derived from its apatite formation capability. Calcium ions in the pulp capping material react with the phosphates in dentin, forming an apatite-like structure with subsequent mechanical and chemical bonds with dentin, this improves the pulp capping sealing ability and presents what is called ‘self-healing restorations’^61^.

In this study, experimental groups were immersed in artificial saliva for 14 days to analyze their apatite formation ability. The shape and size of the created apatite crystals significantly affect the bioactivity of the material. Smaller apatite crystals absorb more protein, thus absorbing more cells capable of inducing hard-tissue regeneration^2^.

As revealed in the SEM images, groups B and C revealed densely formed small crystals, suggesting that these groups can induce cell proliferation and hard-tissue formation^2^.

According to SEM observations and EDX analysis, MTA and the experimental group B, and C exhibited apatite-forming ability while group A did not, obviously due to the absence of portlandite in this group.

Ca/P ratio of calcium phosphate precipitates significantly affects the degree of their bioactivity. Previous studies reported Ca/P ratios of 3.84, 8.33, and 2.74 for MTA incubated in simulated body fluid for 1, 7, and 14 days respectively^68^. In the current work, the Ca/P ratio of the calcium phosphate precipitated in MTA was higher than the stoichiometric Ca/P ratio for hydroxyapatite (1.67). On the other hand, the Ca/P ratio of the precipitates in group B (25% silica and 25% portlandite) has a closed Ca/P ratio to the hydroxyapatite, while the Ca/P ratio in the case of sample C (50% portlandite) was higher than that of hydroxyapatite and close to that of positive control. The high Ca/P ratios may be an indication of the presence of excess calcium precipitations on the surface, which provide favorable bioactivity, biocompatibility, and hard tissue-induction abilities for the experimental restoration materials^2^.

The clinical success of bioactive pulp capping materials mainly depends on their ability to form and regenerate the apatite phase of hard tissues. Their hard tissue-induction abilities are derived from the produced calcium hydroxide resulting from the hydration reaction of MTA^2^.

The results of our study demonstrated that groups B and C pulp capping materials possessed high bioactivity and produced modification in their surface morphology and chemical composition upon immersion in the artificial saliva, equivalent to or even surpassing those of the positive control group (MTA). This significant finding was evidenced by the formation of packed calcium phosphate nano-spherulite clusters with needle-like projections shown in group C. This high bioactivity could be explained by a previous study^63^, which stated that the hydroxyl, ester, and ether chelating groups (OH, C=O, C–O–C, and C–O groups) present in hydroxyl ethyl methacrylate resin (HEMA) are the coordination sites for chelating calcium ions^63^. Likewise, Bis-A GMA and TEGDMA resins found in the experimental groups of the present study could form Bis-A GMA and TEGDMA-calcium chelate complexes. These complexes are responsible for providing initiation sites for apatite nucleation.

Further studies are recommended to evaluate the biocompatibility of these novel experimental pulp capping materials on dental pulp cells and to compare their cytotoxic effect and dentin formation capability to a commercially available MTA product, at different time intervals.

## Conclusion

Within the limitation of this study, it was concluded that a reinforced bioactive resinous pulp capping material could be achieved using low-cost rice husk and carbonated mud from the sugar industry to obtain silica and portlandite nanoparticles respectively and that the produced pulp capping restorations have equivalent bioactivity to MTA but with better mechanical properties and controlled setting time.

## Data Availability

All data generated or analyzed during this study are included in this article.
